# Advances in Single-Cell Transcriptome Sequencing and Spatial Transcriptome Sequencing in Plants

**DOI:** 10.3390/plants13121679

**Published:** 2024-06-18

**Authors:** Zhuo Lv, Shuaijun Jiang, Shuxin Kong, Xu Zhang, Jiahui Yue, Wanqi Zhao, Long Li, Shuyan Lin

**Affiliations:** 1 Co-Innovation Center for Sustainable Forestry in Southern China, Nanjing Forestry University, Nanjing 210037, China; zlv@njfu.edu.cn (Z.L.); 17851686906@163.com (S.J.); konhshuxin@163.com (S.K.); 17645104840@163.com (X.Z.); yjh202312@163.com (J.Y.); wanqzhao@163.com (W.Z.); lilong1949@126.com (L.L.); 2 Bamboo Research Institute, Nanjing Forestry University, Nanjing 210037, China; 3 College of Life Science, Nanjing Forestry University, Nanjing 210037, China

**Keywords:** plants, single-cell transcriptome sequencing, spatial transcriptome sequencing, research progress

## Abstract

“Omics” typically involves exploration of the structure and function of the entire composition of a biological system at a specific level using high-throughput analytical methods to probe and analyze large amounts of data, including genomics, transcriptomics, proteomics, and metabolomics, among other types. Genomics characterizes and quantifies all genes of an organism collectively, studying their interrelationships and their impacts on the organism. However, conventional transcriptomic sequencing techniques target population cells, and their results only reflect the average expression levels of genes in population cells, as they are unable to reveal the gene expression heterogeneity and spatial heterogeneity among individual cells, thus masking the expression specificity between different cells. Single-cell transcriptomic sequencing and spatial transcriptomic sequencing techniques analyze the transcriptome of individual cells in plant or animal tissues, enabling the understanding of each cell’s metabolites and expressed genes. Consequently, statistical analysis of the corresponding tissues can be performed, with the purpose of achieving cell classification, evolutionary growth, and physiological and pathological analyses. This article provides an overview of the research progress in plant single-cell and spatial transcriptomics, as well as their applications and challenges in plants. Furthermore, prospects for the development of single-cell and spatial transcriptomics are proposed.

## 1. Introduction

Cells constitute the fundamental structural and functional entities of living organisms. The intricate processes of growth, development, and differentiation in cells are intricately connected to alterations in the expression of DNA and protein content within organisms [1]. Consequently, investigating organisms at both cellular and developmental levels represents a significant concept and avenue for advancing modern biotechnology. Advances in single-cell separation technology have facilitated the isolation, examination, and juxtaposition of individual cells. During their growth trajectory, cells undergo numerous divisions and proliferations. As they complete their life cycle, they also exhibit cellular diversity in terms of cellular state or cell type, attributable to modifications in gene expression. This phenomenon of diversity is referred to as cell heterogeneity [2]. Cell heterogeneity is universally observed in organisms, wherein cells exhibit distinct shapes and possess specific functions, along with varying gene expression patterns [3].

The transcriptome broadly refers to the collection of all transcription products in a cell under a certain physiological condition, including messenger RNA, ribosomal RNA, transporter RNA, and non-coding RNA, narrowly referring to the collection of all mRNAs [4]. Transcriptome sequencing (RNA-sequencing, RNA-seq) is the technique of sequencing and analyzing all or part of mRNAs, small RNAs, and non-coding RNAs in cells or tissues using high-throughput sequencing technology [5]. Conventional transcriptomics studies usually sequence an entire organ or tissue, and the results correspond to the mean value of gene expression in a group of cells, ignoring the heterogeneity between cells [6]. Although helpful in addressing many biological questions at the organ or tissue level, it is not possible to understand transcriptional processes in rare cell types or individual cells. Single-cell transcriptomes and spatial transcriptomes can fill in the missing parts of conventional transcriptomes, with single-cell transcriptomes providing insight into the transcriptome at the single-cell level and spatial transcriptomes helping to conserve spatial information, all of which address biological questions at the cellular level as well as cellular heterogeneity.

Early studies on single-cell RNA sequencing (scRNA-seq) and spatial transcriptome sequencing focused mainly on animal cells, and relatively little research has been conducted on scRNA-seq and spatial transcriptome sequencing in plants. Currently, scRNA-seq and spatial transcriptome sequencing have become a research hotspot in plants. With the gradual development of scRNA-seq and spatial transcriptome sequencing from the field of medicine to the field of botany, the problems of the genetic heterogeneity and spatial heterogeneity of plant cells are being solved. At the same time, scRNA-seq and spatial transcriptome sequencing still facing great challenges in plants, which have hindered research at the plant cell level. Several papers have been published to provide a better overview of the principles and methods of plant single-cell transcriptomes and some of the research progress in plants [7,8,9], while fewer research reviews have reported on plant spatial transcriptome sequencing. This review aims to delineate and encapsulate the advancements in research regarding plant scRNA-seq and spatial transcriptome sequencing while also offering insights into future developments.

## 2. Advances in Single-Cell Sequencing and Its Study in Plants

Single-cell sequencing amplifies and sequences DNA or RNA from the level of a single cell, which mainly includes single-cell sorting, nucleic acid extraction, library construction, high-throughput sequencing, and data analysis. Single-cell sequencing mainly includes single-cell DNA sequencing (scDNA-seq), scRNA-seq, and single-cell epigenome sequencing, which can reveal the functions and properties of cells at different stages from different perspectives. These three types of sequencing can reveal the functions and properties of cells at different stages from different perspectives. There are two sequencing methods for scRNA-seq, one for sequencing protoplasts and the other for sequencing nuclei, both of which have the disadvantage of losing spatial information about cells when they are isolated from tissues [10].

### 2.1. ScRNA-seq Technology

ScRNA-seq is a new technology for amplifying and sequencing the entire transcriptome at the single-cell level using the basic principle of microRNA amplification of isolated single cells followed by high-throughput sequencing to obtain the expression profiles of individual cells, identify the cell type, and analyze the spatial and temporal course of cell development [11]. ScRNA-seq can provide the complete transcriptome expression profile of a single cell. ScRNA-seq first requires sample isolation to obtain individual cells, and then amplification and sequencing of trace amounts of total transcriptome RNA from individual cells. Early single-cell studies targeted only a small number of valuable cells, but in recent years, improvements in library construction and sequencing in various parts of the process have led to a continuous reduction in cost, the amount of valid information has increased, a single experiment can simultaneously detect tens of thousands of cells, and the efficiency of experiments has been greatly improved [12]. A variety of scRNA-seq technologies have been developed, with different applications, and they are now widely used in medical and animal research, with PCR plate-based and droplet-based technologies being the two most widely used scRNA-seq technologies in plant research [13]. ScRNA-seq has opened up the possibility of understanding transcriptional processes in different cell types or single cells [14]. Compared to animal cells, scRNA-seq has been used less in the study of plant cells because the presence of plant cell walls makes it more difficult to isolate and obtain single cells [15]. However, it also shows great potential in botanical research, and scRNA-seq in plants can help to discover rare cell types in depth, understand the roles of different cell types in the developmental process, and reveal the mechanisms of cellular response to environmental changes.

### 2.2. Advances in ScRNA-seq in Plants

To explore the establishment of allele expression in plant single cells, in 2017, Han et al. developed a scRNA-seq protocol and applied it to rice, reporting the first application of scRNA-seq in plants [16]. Since its introduction, scRNA-seq in plants has been applied to studies in the fields of plant cell mapping and plant organ development. At present, most of the studies on plant single cells are based on cell clustering and differential gene analysis, followed by elucidating cell development and its regulatory mechanisms, and then deeply exploring cell response mechanisms under abiotic stress environments. Due to limitations at the technical level, the species of plant in single-cell research was limited to *Arabidopsis* in the initial stage, but with a breakthrough in the technical bottleneck, it has been gradually expanded to *Oryza sativa*, *Zea mays*, *Brassica rapa* var. *glabra* Regel, *Solanum lycopersicum*, *Populus*, *Arachis hypogaea*, and many other species, involving a variety of tissue types such as roots, stems, leaves, flowers, pollen, seed, endosperm, and so on. The research progress of scRNA-seq/snRNA-seq and spatial transcriptome sequencing in plants is shown in Table 1.

#### 2.2.1. Advances in ScRNA-seq in Plant Roots

Roots are the nutrient organs of plants, with the roles of support, reproduction, and storage for synthesizing organic materials, and scRNA-seq studies of the mesocarp are mainly focused on the development of the root system. In 2019, Zhang et al. used a combination of high-throughput scRNA-seq and cell-type marker-based cell sorting to study *Arabidopsis* roots, finding that the cellular transcriptome in *Arabidopsis* roots is highly heterogeneous. The hierarchical structure of roots was dissected through the spatial distribution and temporal order of individual cells, and the successive differentiation trajectories of root development were reconstructed. A total of 24 hypothetical cell clusters and cluster-specific marker genes were also identified in roots, and each root cell cluster showed different patterns of ion assimilation and hormonal responses, among which growth hormone-related factors *IAA33*, *ARF5/10*, and *YUC3/8/9* were highly expressed in meristematic tissues, in addition to *PLT1* and *RGF2/RGF3* [17]. In the same year, Tom et al. sequenced *Arabidopsis* root tissues using scRNA-seq, obtained gene expression data using conventional transcriptome sequencing methods, and correlated the results with those of scRNA-seq to map the high-resolution gene expression profiles of *Arabidopsis* root tissues, identifying the major cell types as well as the scarce cells in quiescent centers, and found that *ATL75* was highly expressed in the mesophyll, *MES15* was highly expressed in differentiating trichoblasts, *PIP2-8* was highly expressed in the meristematic vasculature, and *TEL1* was highly expressed in root quiescent centers [18]. Later, some researchers performed scRNA-seq on the first four stages of lateral root formation in *Arabidopsis*, which revealed the ontological level of lateral root formation [27]. In 2022, Shahan et al. analyzed 96,000 cells from *Arabidopsis* seedling root tips by scRNA-seq, combined with published data related to single-cell sequencing, constructing the first spatiotemporal expression profile of 100,000 root cells at the single-cell level, and deduced the developmental trajectory of the root cell lineage. The highly expressed genes in major cell types were analyzed. For instance, *SMB* and *BRN1* in the xylem, *TEL1* in the quiescent center, *XPP* in the xylem pole pericycle, *SHY2* and *MYB36* in the procambium and endodermis, *COBL9* in the trichoblast, and *GL2* in the atrichoblast were analyzed [19]. In 2020, Liu et al. drew the first single-cell resolution transcriptome map of the root tissue of the monocotyledonous model plant, rice, by scRNA-seq, which provided a theoretical basis and genetic resources for the study of the rice functional genome [27]. Sequencing analysis of the rice radicle using a combination of ATAC-seq and scRNA-seq and the cellular maps drawn from single-cell sequencing analysis of rice embryonic roots depicted the differentiation trajectories of rice root epidermal cells and basic tissue cells, elucidating the regulatory mechanisms of transcription factors for cellular differentiation. Analysis of the transcriptome profiles and marker genes of *Arabidopsis* root tips and rice root tips revealed the conservatism and differentiation of root developmental pathways in dicotyledonous plants and monocotyledonous plants. It was also found that *bHLH*, *bZIP*, and *GATA* were highly enriched in the meristematic zone, and *MYB12* and *MYB52* were highly expressed in the elongation zone region. Root conformation contributes to plant adaptation to the environment, and the comprehensive definition of cell types by scRNA-seq contributes to the understanding of root conformation, which lays the foundation for understanding root cell differentiation and adaptation of root architecture to environmental factors [28]. High-throughput scRNA-seq was performed on the apical tissues of *Phyllostachys edulis* basal root, and the captured single cells of bamboo basal root could be categorized into 13 different cellular taxa. It was found that the root crowns and epidermis of bamboo originated from a common initial cellular lineage, which was different from that of the monocotyledonous model plant, rice, and the developmental specificity of bamboo basal root was revealed. Transcription factors *PheWOX13a* and *PheWOX13b*, specifically expressed in the root crown taxa of bamboo, were also found to participate in the developmental process of plant primary roots, lateral roots, and root crowns, which provided new insight into the involvement of *PheWOX13* in the development of roots. The first study on the unfolding of bamboo rattan root conformation using scRNA-seq provided a theoretical basis for the study of bamboo plant adaptation to environmental factors, which laid a certain foundation for the development of the bamboo industry [36]. The root tips of the leguminous plant, *Lotus japonicus*, were identified by combined scRNA-seq and bulk RNA-seq analysis identifying seven cell clusters corresponding to seven major cell types, and analysis of the highly expressed genes in each cell type revealed that *Lj1gvBR110561* was expressed in the cortex, *Lj1gvBRI21026* was expressed in the middle of the cortex, and *Lj1gVBR121766* was expressed in both the root cap and root hairs. Further analyses by in situ hybridization validation revealed the phytohormone and plant growth regulatory networks associated with specific cell types and revealed the conserved and divergent features of the cell types. This study was the first single-cell resolution transcriptome study of leguminous root tips and provides a good basis for the study of the development and physiological functions of various cell types in leguminous plants [37].

#### 2.2.2. Advances in ScRNA-seq in Plant Stems

The stems of plants play transport and support roles, and scRNA-seq of stems has focused on the regulation of stem meristem differentiation and spatiotemporal developmental trajectories. Tian et al. performed single-nucleus RNA sequencing (snRNA-seq) and bulk RNA-seq combined analysis on tomato stem tips, identified major stem tip tissues and different cell types at developmental stages, mapped the developmental trajectories of chloroplasts, the vasculature, epidermis, and trichomes, and constructed the spatiotemporal gene expression profiles of heterogeneous stem tip cells. The differential genes in the major cell types were also analyzed, and the major differential genes are shown in Table 2 [51]. Transcriptional profiles of stem cell niches and their differentiated daughter cells in maize stem tips were obtained after scRNA-seq analysis of maize stem tips, and it was found that stem cells located in stem tip meristematic tissues would ensure genome integrity and had low cell division rates, consistent with their contribution to acting on germline and somatic cell fates. It was also found that ectopic expression of *KN1* accelerated cellular differentiation and facilitated maize sheath-leaf base development [32]. In 2021, Zhang used the 10× Genomics scRNA-seq platform with droplet-based scRNA-seq technology to sequence *Arabidopsis* stem tips, found that the stem tips were composed of highly heterogeneous cells with 23 cell clusters, and also described the developmental trajectories of epidermal cells, vascular tissues, and chloroplasts. Analysis of differential genes during stomatal development revealed that *POLAR*, *BASL*, and *TMM* were highly expressed in the early stage of development. *POLAR* and *BASL* were highly expressed in the S stage, while *EMM* and *EPF2* were highly expressed in the M stage or the G0/G1 stage. With the development of stomatal pores, *MUTE*, *FMA*, and *MYB60* were progressively highly expressed with the common and distinctive features of the epidermis and vascular tissues. The study of stem tip stomata at single-cell resolution provides a sufficient theoretical basis for the basic principles of plant cell division and differentiation [21]. ScRNA-seq was performed on the xylem of 5-month-old poplar seedlings, 9798 isolated cells were divided into 12 cell clusters, and vascular cells, fibroblasts, ray parenchyma cells, and xylem precursor cells were identified by in situ hybridization. The cell differentiation trajectories indicated differentiation processes for vascular and fibroblasts and similar differentiation processes for fibroblasts and ray parenchyma cells, and marker genes and key candidate regulators for each cell type in xylem cell differentiation were identified. It was suggested that *PagSND2* and *PagMYB42* may be important regulators of vascular differentiation, and *PagSND1* may be a key regulator in fibroblast differentiation [38]. Subsequently, Chen et al. also studied the xylem of 4-month-old poplar seedlings, in which 20 cell clusters with a high degree of heterogeneity were identified, with different clusters showing different patterns of phytohormone responses, and the cellular differentiation trajectories of phloem and xylem development were reconstructed. It was found that *SMXL5* and *OPS* were significantly expressed in phloem fulcrum and adjacent cells, and *HD-ZIP III* transcription factors *PtrHB4*, *PtrHB7*, and *PtrHB8* played important roles in regulating vascular cambium activity and xylem development in poplar [39]. Single-cell studies of poplar xylem have established the transcriptional profiles of the major cell types of poplar stems at single-cell resolution, providing a theoretical basis for studying the fundamentals of vascular cell differentiation in trees.

#### 2.2.3. Advances in ScRNA-seq in Plant Leaves

Leaves are the main organs of photosynthesis and respiration in plants, and scRNA-seq of plant leaves is mainly focused on respiration, photosynthesis, and development. In 2020, Sun et al. analyzed the scRNA-seq data of 12,844 cells obtained from the cotyledons of 5-day-old *Arabidopsis* seedlings, and, for the first time, used scRNA-seq to resolve the transcriptome dynamics pattern of the development process of *Arabidopsis* stomatal cells. Most of the 11 identified cell clusters corresponded to cells at specific stomatal developmental stages. Potential interactions between transcriptional networks and genes regulating the development of meristematic tissues to guard mother cells were also revealed. Analysis of genes in different developmental stages of the stomata revealed that *WRKY33* and *BPCs* were involved in the regulation of early stomatal development; *EPF1*, *SCRM/2*, *FAMA*, and *HIC* were highly expressed in guard mother cells; *FAMA*, *HRBCS*, and *EPF1* were highly expressed in early guard cells, and *FAMA*, *HIC*, and *RBCS* were expressed in mature guard cells [22]. ScRNA-seq of 14,117 single cells from 3-day-old *Arabidopsis* cotyledons identified 10 cell clusters and their developmental trajectories, discovered several new marker genes and developmental features of leaf veins as well as cell types, and identified new leaf vein regulators (companion cell: *HIPP36*, *AHP1*, *TET6*, *PHL12*; xylem parenchyma: *AGP31* and *CEL1*; sieve tube elements: *DOF2.4*; phloem parenchyma: *IRX7*, *CDF4*, *SULTR2;1*, *BZIP9*), as well as possible roles for *CDF5* and *RGA* in the early development and function of cotyledonary leaf veins [23]. ScRNA-seq of the vascular system of 6-week-old mature *Arabidopsis* leaves generated a single-cell transcriptome profile of the leaf vascular system, identifying at least 19 cell clusters and clarifying the functional roles played by the various types of cells and the metabolic pathways they are involved in, which provided information on the leaf vascular system as well as the roles of the cell types of the leaf and demonstrated that *bZIP9* promoter activity is specific to phloem parenchyma cells in leaf vasculature. This study provides a key resource for developing strategies to influence ion, metabolite, and signaling fluxes [24]. By analyzing 13,000 stomatal lineages of *Arabidopsis* leaves, marker genes in different types of cells were used to define vascular tissue, mesophyll, and epidermal cell clusters, and the specific genetic programs of these cell types as well as the polarity characteristics of the adaxial/abaxial axis surfaces of leaves were revealed through comparative analysis of cell identity and locus. Fateful differentiation trajectories toward stomatal fate that were previously characterized only by cell morphology were identified, and it was confirmed that the initiation and termination of protective cell differentiation were regulated by the transcription factors *GATA2* and *HAT5*, respectively [25]. ScRNA-seq analysis of peanut leaves constructed cellular differentiation trajectories in peanut leaves, indicating significant temporal heterogeneity in the developmental process of peanut leaf differentiation, revealing the mechanism of formation of chloroplasts, and inferring the mechanism of epidermal cell formation. It was also deduced that the mechanism of epidermal cell formation was closely related to the expression levels of transcription factors. In this study, it was also found that *AHL23* was localized in the nucleus and ectopically expressed in *Arabidopsis*, which promotes leaf growth by regulating phytohormone pathways, and that the application of scRNA-seq would provide new hypotheses for the cellular differentiation of the leaf cells of the heterotetraploid peanut and other plants [40]. ScRNA-seq data of maize leaves were analyzed to identify co-regulatory modules through the proposed temporal sequence data obtained from gene regulatory network analysis, demonstrating that several TF families play an important role in regulating maize M-cell development and are involved in epigenetic regulation. Among them, *WRKY*, *ERF*, *NAC*, *MYB*, and *HSF* families were highly expressed in early M-cell development, with *HSF* playing a dominant role, and *HSF1* was expressed at the leaf base, while *COL* and *ERF* families were highly expressed in later stages of M-cell development, with *COL* playing a dominant role, and *COL8* was expressed at leaf tips [33]. Protoplasts prepared from young leaves of Chinese cabbage were sequenced, and 16,055 high-quality cells were divided into 17 cell clusters. Eight cell types, namely mesophyll, epidermis, vascular system, vascular sheath, guard, proliferating, phloem, and xylem cells, were identified using direct homologs of marker genes in *Arabidopsis* [31]. The cells isolated from young leaves of cassava (*Manihot esculenta*) contained 15 cell clusters, and the three major tissue types in cassava leaves, namely, chloroplasts, epidermal cells, and vascular tissues, were successfully identified using marker genes from *Arabidopsis*, with chloroplasts expressing photosynthesis-related genes (*CHS*, *LHCA5*, *PSBP*, *YAB1*), epidermal cells expressing wax and cork-related genes (*YAB1*, *RBR*, *TIR1*, *AFB2*, *H2B-3*), and vascular cells expressing amino acid transporter genes (*DOF5.6*, SEOB, SUC2, NAKR2, *ACL5*, *COBL4*). Analyzing the structure and photosynthetic properties of cassava leaves at the single-cell level can provide a theoretical basis for further improving cassava yield and nutritional quality [41]. ScRNA-seq of tea tree (*Camellia sinensis*) leaves constructed a transcriptome map of leaf cells, mapped the developmental trajectories of leaf cells, and identified the distribution of different cell types during leaf differentiation and genes related to cell fate transformation. Genes involved in the biosynthesis of certain metabolites showed cell-specific and developmental specificity, and tea catechin ester glycosyltransferases were also characterized for the first time in plant chloroplasts through gene co-expression networks, providing evidence that *UGT72B23* may be a marker gene for sponge tissue [42]. The transcriptome of rubber tree (*Hevea brasiliensis*) leaves during early powdery mildew infection was characterized using scRNA-seq, identifying 10 cell types and constructing the first single-cell atlas of rubber tree leaves under powdery mildew infection, in which gene expression patterns were observed in different cell clusters. It was also found that the repetitive protein encoding the nucleotide-bound leucine-rich protein of *HbCNL2* regulates rubber leaf defense against powdery mildew, and overexpression of *HbCNL2* causes cell death in tobacco leaves and elevated levels of reactive oxygen species in rubber tree leaf protoplasts. This study was the first to observe the cellular and molecular responses of rubber tree leaves to infection by biotrophic pathogens, a finding that provides a valuable basis and resource for the identification of disease resistance genes in important tree species [43]. The first single-cell atlas of metabolism in the leaves of *Taxus* was established with 8846 cells isolated from the leaves of *Taxus* and divided into 15 cell clusters, which revealed the spatial and temporal expression patterns of several secondary metabolic pathways and also demonstrated that the cells of *Taxus* leaves have a high degree of heterogeneity. Many novel and cell-specific transcription factors involved in secondary metabolite biosynthesis have also been identified, including *MYB17*, *WRKY12*, *WRKY31*, *ERF13*, *GT_2*, and *bHLH46.* The identification of the main cell types in *Taxus* leaves at single-cell resolution provides a theoretical basis for studying the basic principle of cell type-specific regulation of secondary metabolism [44].

#### 2.2.4. Advances in ScRNA-seq in Plant Flowers

Flowers are the reproductive organs of plants, and scRNA-seq studies on flowers have focused on inflorescence differentiation and meiosis. Brad Nelms and Virginia Walbot’s study of maize germ cell meiosis by scRNA-seq revealed that altered nuclear events and cellular morphology correlate with altered transcriptional expression during meiosis, with a loss of ribosomal transcripts and an increase in transcripts of membrane-bound organelles, and that the cytoplasmic remodeling of meiosis correlates with cell transformation from diploid to haploid [34]. Using maize ears as the research object, 12,525 single-cell expression profiles were obtained by scRNA-seq, and 12 cell clusters were constructed, which provided important basic data for maize basic research and breeding. This study could predict the redundancy of the maize TPP family controlling inflorescence branching and identify the delayed flower transition phenotype of a redundant maize *VOZ* family through multiple *CRISPR-Cas9* mutagenesis, which provides an important data basis for maize basic research and breeding [35]. Single-cell transcriptional data analysis of *Nicotiana attenuata* corolla cells identified 3765 corolla cells and, by comparing the expression patterns of different genes among the 3765 cells, key genes (*Na4CL1*, *NaPKS2* and *NaAER1*) involved in the benzylacetone (BA) synthesis pathway were identified. It was confirmed that BA is predominantly synthesized within the epidermal layer of corolla cells [46]. A single-cell dynamic panorama covering the transition from inflorescence to floret during early reproductive development was constructed by analyzing the rice inflorescence through the single-cell transcriptome, and molecular and genetic analyses demonstrated that the *WOX* transcription factor, *DWARF TILLER1*, functions in regulating the activity of the rice floral meristem tissue and also confirmed the role of the growth hormone transporter protein, *OsAUX1*, in the development of inflorescence, providing evidence for the role of growth hormones in rice inflorescence meristems [29].

#### 2.2.5. Advances in ScRNA-seq in Plant Fruits/Seeds

Single-cell transcriptome studies of fruits/seeds have focused on endosperm development, cell differentiation trajectories, and characterization of gene expression dynamics during reproduction. Such tissues are difficult to dissociate from protoplasts, and nucleus lifting is generally preferred. SnRNA-seq of *Arabidopsis* seeds yielded a high-quality transcriptome of 1437 endosperm nuclei, confirming that *Arabidopsis* endosperm contains an undescribed differential transcriptional diversity of nuclei, and determining for the first time that gene imprinting is dynamic throughout the cell cycle and that a subset of imprinted genes are heterogeneous among cell or nucleus types. This study contributes to the understanding of epigenetic influences on crop seed development [26]. scRNA-seq of the *Bombax ceiba* ovary resulted in the identification of a total of 15,567 high-quality cells from the inner wall of the cotton ovary, the identification of 347 potential marker genes for the fiber initiation cell type, and the identification of two major cell types, fiber initiation cells and epidermal cells, by RNA in situ hybridization. As well as the major marker genes in the two cell types, *PDF1* in initiating fiber cells and *HIC* in epidermal differentiation, the study of *B. ceiba* fiber initiation contributes to the improvement of cotton fiber yield [47]. In 2023, Lee et al. collected more than 800,000 *Arabidopsis* nuclei located at different developmental stages and in different organ tissues for snRNA-seq and constructed a single-nucleus transcriptional atlas of *Arabidopsis* between the seed and the production of new seed, which provides a resource for the study of the characterization of the cell types of the plant throughout its development [52]. ScRNA-seq of mature ovaries of two oil tea (*Camellia oleifera*) varieties with different rates of ovule septation yielded 20,526 high-quality cells, identified 18 cell clusters, and characterized six cell types, including female gametophyte, primary xylem, primary phloem, procambium, epidermis, and parenchyma cells, from the three major tissue types of the ovule, placenta, and endocarp. Comparative analysis of high- and low-OAR varieties demonstrated significant upregulation of the overall expression of *CoSWEET* and *CoCWINV* in cells of the procambium of the low-OAR variety, as well as *CoSTP* in the pericycle. Meanwhile, the authors also proposed that ovule abortion may be related to sugar transport in the placenta and ovule. This study reports for the first time a single-cell transcriptional landscape in the ovary of a woody crop, providing new insight for further deciphering the mechanisms of sugar transport regulation and seed yield enhancement in *C. oleifera* [48]. Using scRNA-seq technology, 14,535 cells were identified from the outer ovule of *Gossypium hirsutum* at different stages of development, three main cell types were identified (fiber, non-fiber epidermis, and outer pigment layer), and the development trajectory of the outer ovule fiber cells was reconstructed. The gene regulatory network revealed that *MYB25-like* and *HOX3* play key guiding roles in fiber differentiation and tip-biased diffuse growth, respectively [49]. Wang et al. performed a combined analysis of scRNA-seq and single-cell ATAC sequencing (scATAC-seq) in the extragalular coat cells of two cotton species, identified five cell populations, and constructed the developmental trajectory of fiber lineage cells, demonstrating that the primary growth of fiber cells is a highly regulated circadian process. Regulation of circadian metabolism and protein translation in mitochondria by CREs, TCP motifs, and TCP-like motifs in conjunction with the trans factor GhTCP14s regulated circadian metabolism and protein translation in roughly one-third of the highly expressed genes in fiber cells, revealing a new pathway for improving fiber traits by modifying the biological clock of fiber cells [50].

## 3. Advances in Spatial Transcriptome Sequencing and Research in Plants

Spatial transcriptome sequencing is based on scRNA-seq technology that preserves and records the original spatial location of cells, providing information on the “spatial location of organization” of gene expression and revealing the spatial heterogeneity of cells. Spatial transcriptomics combines tissue imaging and RNA sequencing to simultaneously quantify and localize gene expression. Spatial transcriptomics is performed by placing frozen sections on arrays with reverse-transcribed oligonucleotide (dT) primers containing unique positional barcodes, and the sequencing process involves section fixation, staining, and permeabilization, followed by localized capture and reverse transcription of the mRNA, removal of the tissue, release of mRNA-cDNA hybridization, and finally, combining the morphology of the tissue sections with the barcoded gene expression data.

### 3.1. Spatial Transcriptome Sequencing Technologies

Spatial transcriptomics techniques originated in mammalian systems and have been widely used in mammals, with less research in plants. The study of spatial transcriptomics in plants mainly focuses on finding ways to utilize and optimize spatial transcriptomics methods, as well as using spatial technologies to solve scientific problems in plant research [10]. In spatial transcriptomics sequencing, some high-throughput in situ gene expression analysis methods have been developed [50,67,68,69,70], such as Slide-seq [71], DBiT-seq [70], and the 10× Genomics Visium platform [72]. Most of these methods have been successfully applied to mammalian studies and have yielded many new insights into development and disease progression [68]. However, so far, only one method has been applied to plant systems, and spatially enhanced resolution histological sequencing (stereo-seq) is, so far, the only sequencing-based transcriptomics technology with spatial resolution at subcellular resolution [67]; therefore, technical difficulties in plant tissue preparation remain a challenge [55].

ScRNA-seq is currently unable to provide high-resolution spatial transcriptomic information or identify subcellular organoids in biological samples. These limitations have led to the development of spatially enhanced resolution histology sequencing, which combines spatial information with single-cell transcriptomics to address the challenges of using scRNA-seq alone [73]. Spatial transcriptome sequencing combines single-cell information with high-resolution spatial transcriptomics to provide spatiotemporal transcriptome information at the subcellular level, maintaining high sensitivity while also capturing spatiotemporal transcriptomes from small samples and providing cellular subtyping information from biological samples. In plants, the first use of single-cell combined spatial transcriptomics was applied to a leaf sample of the model plant, *Arabidopsis*, to generate an in situ transcriptome [65].

### 3.2. Advances in Spatial Transcriptome Sequencing in Plants

Spatial transcriptome-related research has been mainly focused on the medical field and relatively little research has been performed in plants compared to scRNA-seq. However, studies on stems, leaves, flowers, fruits/seeds, and healing tissues of plants have been reported. In addition, some studies have utilized a combination of single-cell and spatial transcriptome approaches to address the problem of cellular heterogeneity while preserving the spatial location information of tissues.

Study of the plant spatial transcriptome first began in 2017, when Giacomello et al. used three tissues, *Arabidopsis* inflorescence meristem, European *Populus tremula* leaf buds, and *Picea abies* female cones, to apply the spatial transcriptome, presented three important results: experimental feasibility, consistency of tissue characteristics, and experimental stability. Although the article did not address specific biological aspects of the three tissues, it opened a whole new door for spatial transcriptome research in plants [55]. In 2018, Giacomello et al. continued the exploration of spatial patterns in more gymnosperm and angiosperm transcriptomes, providing different treatments for cell walls of different plant types using different enzyme mixtures, as well as plant tissue freezing and permeabilization optimization and tissue removal methods, while the highly heterogeneous tissue sites of *Arabidopsis* inflorescence, European *P. tremula* leaf buds, and *P. abies* female cones, in particular, accurately improved the specificity (93%), accuracy (71%), and sensitivity (60%) of spatial gene detection in plants [56]. In 2020, Stefania Giacomello and Jens Sundström combined 10× Visium technology to further investigate spatial gene expression patterns in female reproductive development of wild-type and early conical mutants of Norway *P. abies* to characterize the spatial development of *P. abies* female cones at higher resolution [57]. Spatial transcriptome sequencing of ovules and ovary wall/pericarp at four different developmental stages of early tomato fruits provided a full picture of the spatial distribution of signals in the early stages of ovule and pericarp development, the results of the study showed that hormonal signals were initiated in ovules and pericarp after fertilization and different signals were activated due to the different developmental processes of ovules and pericarp. For example, *PIN5* is specifically expressed in ovules and expression levels gradually increase after fertilization, whereas *PIN6*, *PIN1*, *PIN7* and *LAX3* are mainly expressed in the ovary wall/pericarp and expression decreases after fertilization [58].

In 2019, Michael Giolai et al. used GaST-seq technology to detect spatial gene expression characteristics in eight regions of *Arabidopsis* leaves to explore gene stress expression as well as spatial-specific gene expression in leaf tissues. Sequencing found that there were 393 differentially expressed genes in the midvein and leaf veins, and 686 differentially expressed genes in the leaf margins and leaf veins. They used the bacterial molecule flagellin-flg22 to infect different tissue regions of leaves, and it was found that the number of differentially expressed genes was not the same in different regions after infection. These genes were mainly enriched in biological processes related to stress and defense responses [54]. Genome-wide transcriptome and spatial transcriptome sequencing analyses were performed on different regions of cabbage plants from the inner leaf to the outer leaf. Through spatial expression analyses of key genes for leaf development and sugar metabolism, the key transition leaf was identified as the first inwardly curved leaf, and it was found that the key transition leaf was controlled by a complex signaling network of internal hormones, protein kinases, external light, and other stimuli. The analysis of the spatial transcriptome of cabbage leaves provides a new idea for the study of leaf heading and new insight for the identification of key transition leaves of cabbage [60]. Mapping and visualizing the expression of genes involved in the metabolism of C4 and sestamibiic acid at the spatial level in *Portulaca oleracea* and spatially explicit analysis of gene expression indicated that the C4 and CAM systems are fully integrated in *P. oleracea*, that carbon fixation by CAM and C4 occurs in the same cells, and that metabolites produced by CAM may be directly incorporated into the C4 cycle. This study demonstrated that metabolic engineering of C4+CAM in C4 crops was feasible and can lead to substantial drought resistance in crops with unchanged yields [62].

By applying 10× Visium spatial transcriptome technology to study the developmental process of floral organs in the orchid *Phalaenopsis* Big Chili, multiple cell types of early orchid development were identified through the analysis of high-resolution spatial expression patterns of key genes of floral development, including inflorescence meristematic tissues, floral primordia, multiple floral organ primordia, and multiple meristematic tissues, in which the meristematic tissue cells at the base of the floral organ continued to function at multiple developmental stages after organ initiation. With the development of anthers being the most specific, the MADS-box gene and many other downstream regulators together regulate the differentiation of its progenitor from a single point to a later stage of differentiation into multiple cell types. The trajectories for the differentiation of multiple cell types at different developmental stages were constructed [63]. By combining high-resolution anatomical analysis and spatial transcriptome sequencing of primary and secondary growth of poplar stems, cell type-specific gene expression was localized to specific anatomical domains. The characteristics and specific marker genes of all major cell types in the developmental gradient from primary to secondary vascular tissues of poplar stems were defined, new specific marker genes, such as *CYCP3*, *LRRK*, and *ERF5/017/105*, were identified in meristematic tissues, and the successive process of the gradual development of poplar stems from apical pro-former stem cells to secondary vascular stem cells was traced. This study provides data support for research on vascular tissue development, wood formation mechanisms, and terrestrial vascular plant evolution [64]. Spatial transcriptome sequencing of tomato seed-callus induction revealed, for the first time, the cellular heterogeneity within tomato callus, resolved the expression of hormonal signals and important regulators in various tissues and cells at the single-cell level, analyzed and discovered the subtypes and functions of epidermal and bud progenitor cells, and the expression of *SlPLT3*, *SlWUS*, *SlSTM*, and *SlCUC1* was detected in tomato stem primordia, suggesting that they play conserved roles in stem regeneration in different species. The study revealed the role of chlorenchyma cells in position determination, that differentiation development of bud progenitor cells plays an important role in position determination and differentiation development, and how light drives the bud regeneration process by promoting the development of green tissue cells. This study provides new ideas and insight for molecular breeding efforts in tomato [59].

In 2022, Xia et al. sequenced the single-cell transcriptome and spatial transcriptome of *Arabidopsis* leaves and successfully described the transcriptome differences between the upper and lower epidermis of leaves. Using high-resolution localization information, they found the expression changes of cell type-specific genes in the leaves from the main leaf veins to the margins of leaves, and by reconstructing the expression differences of the genes in the space, they demonstrated the spatial development of vascular cells and guard cells. The cell type-specific characterization in this study provides new insight and resources for the mechanisms of leaf development and its response to environmental stimuli [65]. Combined single-cell and spatial transcriptome analyses of the formation layer during primary and secondary growth of poplar revealed the transcriptome profiles of primary and secondary growth tissues of poplar stems, and the comparison of cell profiles with the differentiation trajectories of primary and secondary growth revealed the different regulatory networks involved in the differentiation process from the formation layer to the xylem precursors and phloem precursors. This study provides new insight into the cellular identity and differentiation that occurs during primary and secondary trunk growth, contributing to the understanding of the dynamics of cellular differentiation during tree stem growth [66]. 

## 4. Experimental Process and Bioinformatics Analysis of ScRNA-seq and Spatial Transcriptome Sequencing

### 4.1. Experimental Procedure for ScRNA-seq

**Figure 1:**
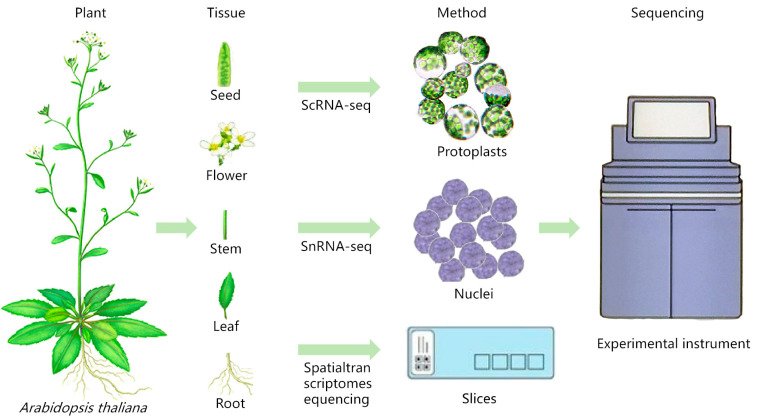
Sample preparation for scRNA-seq/snRNA-seq and spatial transcriptome sequencing of *Arabidopsis thaliana* (refer to Plant Cell Marker Data Base for plant pictures).

ScRNA-seq can be performed by obtaining single-cell suspensions by means of protoplasts or single-cell nuclear suspensions by means of nucleus pumping, as shown in Figure 1. The experimental steps are as follows, using the 10× Genomics sequencing platform as an example: (1) Single-cell (nuclear) suspension preparation: choose fresh plant samples or frozen samples for the experiment. Fresh plant samples can be used for the preparation of protoplasts, while frozen samples can be used for the preparation of single-cell suspensions by means of nucleus extraction. (2) Cell quality check: take an appropriate amount of single-cell suspension, stain it with trypan blue dye, count the cells and calculate the proportion of live cells to ensure that the proportion of live cells is ≥90%, and adjust the cell concentration to the ideal concentration. (3) 10× Genomics single-cell isolation: short read-length sequencing and microfluidics are used to realize simultaneous transcriptome expression profiling of sample cells, and the droplet method is used to combine the gel beads containing the barcode information with the mixture of cells and enzymes to form GEMs. The GEMs flow into the reservoir and are collected, and the GEMs dissolve to release the barcode information. The gel beads are dissolved to release the barcode sequence, reverse transcription is performed to generate cDNA fragments, and the sample is labeled. The gel beads are crushed, the oil droplets are broken up, and PCR amplification is performed using the cDNA as a template. The products of all GEMs are mixed to construct a standard sequencing library. (4) Sequencing library construction: cDNA is enzymatically broken into fragments of about 200~300 bp, and together with the library construction process of traditional second-generation sequencing, such as sequencing junctions and sequencing primers, PCR amplification is performed to obtain a DNA library. (5) Library sequencing: high-throughput sequencing of the constructed library is performed using the double-end sequencing mode of the scRNA-seq platform.

### 4.2. Experimental Procedure for Spatial Transcriptome Sequencing

The main steps of the spatial transcriptome sequencing analysis process include sample preparation, tissue optimization, library construction, on-line sequencing, and data analysis. In order to ensure the integrity of the sections, it is necessary to use isopentane and a liquid nitrogen bath to freeze fresh tissues and OCT embedded frozen fixed tissue. Frozen sections are sliced using a frozen sectioning machine, and tissue sections are collected for RNA extraction for quality control. The frozen sections are affixed to tissue-optimized slides according to region, and the tissue sections are subjected to methanol fixation, H&E staining and bright field imaging, permeabilization and fluorescently labeled cDNA synthesis with different time gradients, and fluorescence imaging. The optimal permeabilization conditions are determined by fluorescence signal intensity and dispersion for subsequent experiments of gene expression library construction. Then, cDNA amplification, library construction, up-sequencing, and data analysis are performed.

### 4.3. Bioinformatics Analysis of ScRNA-seq and Spatial Transcriptome Sequencing Data

**Figure 2:**
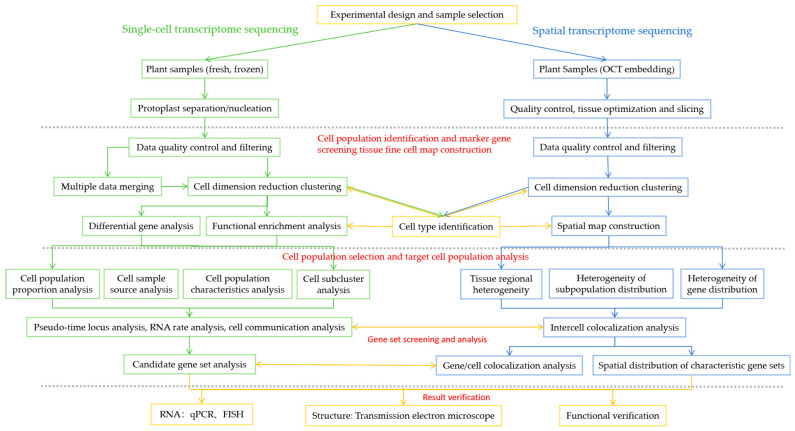
Single-cell sequencing and spatial transcriptome sequencing bioinformatics analysis processes.

The bioinformatics analysis process is shown in Figure 2. For the sequencing data obtained, software is used to obtain single-cell/spatial gene expression data, and third-party software is used to perform downstream analyses, such as single-cell transcriptome/spatial transcriptome subgroup analysis, differentially expressed gene analysis, GO/KEGG functional enrichment analysis, transcription factor prediction, and protein–protein interactions (PPI) network analysis.

#### 4.3.1. Sequencing Data Quality Control and Gene Expression Quantification

The R package Seurat [53] is used to perform data quality statistics on the raw data and to compare the reference genome, gene expression quantification, and effective cell/spot filtering. Single-cell/spot raw data from sequencing and H&E or toluidine blue stained images of spatial tissue sections are used as input data. Using Seurat, cells/spots are further filtered using multiple metrics to retain high-quality cells/spots, reference genome comparison, cell/tissue assay, and spatial barcode/UMI statistics are performed to generate cell/spot gene expression matrices. The median genes per cell/spot, reads mapped to the genome, reads confidently mapped to the transcriptome, and spatial distribution maps of tissue sections are used to perform single-cell/spot spatial mapping, creating distribution maps for quality assessment of single-cell/spatial sequencing data.

#### 4.3.2. Cell/Spot Dimensionality Reduction, t-SNE Visualization, and Cluster Analysis

Seurat-based PCA analysis is used to downscale the data, evaluate the number of genes identified in the cells/spots, the total number of UMIs, and the proportion of mitochondrial gene expression, remove low-quality cells, merge the data, and correct for batch effects using Harmony [45]. The merged data are subjected to clustering, t-SNE visualization, and cell type identification. Seurat clusters the cells/spots using a graph theory-based approach, which classifies all cell/spot clusters into cellular subpopulations to facilitate subsequent analysis. Seurat uses the nonlinear downscaling method, t-SNE, to map high-dimensional cellular data into a two-dimensional space, clustering cells with similar expression patterns to more intuitively visualize intercellular variability [30]. Cell annotation is then performed to identify cell types based on the similarity of the expression patterns of the cells to be identified and the reference cell types.

#### 4.3.3. Gene Differential Expression Analysis

Different cell subclusters are analyzed for differential gene expression using Seurat’s rank sum test [20] to screen for genes with upregulated expression in each cell subcluster. According to the gene expression in each cluster, the differentially expressed genes between different clusters are identified, and different cluster marker genes are identified and differentiated between different clusters. These marker genes can provide basis clues for subsequent identification of the type and function of the tissue region. The clustered heatmaps, bubble maps, violin maps, and spatial distribution maps of the expression changes of different genes in each cluster are visualized to screen the marker genes of cluster clusters.

#### 4.3.4. Functional Enrichment Analysis

The obtained differential genes are compared to obtain the annotation information of the genes, and the GO and KEGG databases are used to perform functional enrichment analysis of the differential genes to speculate the function of the cluster or to assist in the identification of the cell/tissue region type. Differential gene–protein interaction network analysis is performed using String v9 [61], and interaction network maps are constructed using cytoscape.

#### 4.3.5. Pseudo-Time Trajectory Analysis

Pseudo-time analysis, also known as cell trajectory analysis, sorts single cells along a trajectory based on the similarity of expression patterns between sequenced cells as a way of inferring the differentiation trajectories of cells during development or the evolution of cell subtypes. Single-cell trajectories use a matrix of cells and their gene expression profiles are constructed by Monocle. Cells are aligned so that their trajectories can be visualized in reduced dimensional space. Key genes associated with developmental and differentiation processes are identified, and genes with similar expression trends are grouped together in groups that may share common biological functions and regulatory factors.

#### 4.3.6. Personalized Analysis of Data

GSVA analysis [74] can effectively make up for the lack of effective information of microefficiency genes in traditional enrichment analysis and more comprehensively explain the regulation of a functional unit. GSVA analysis reflects the pathway information overexpressed by a subpopulation of cells relative to all cells. Cell cycle analysis: a cell cycle score is calculated for each cell using the R package Seurat based on the expression of marker genes [75] for each cell cycle. RNA rate analysis: RNA rate analysis is performed using the software velocyto (velox + κύτος, quick cell) [76] and embedded in t-SNE plots obtained by downscaling with Seurat using arrows, which are used to demonstrate the differentiation trends of cells.

## 5. Application of ScRNA-seq and Spatial Transcriptome Sequencing in Plants

### 5.1. Mapping Plant Cells

The creation of a plant cell atlas offers invaluable insight into the diverse cell types within tissues, their compositions, and variations in response to environmental cues, developmental stages, and various treatment modalities. It can be used to obtain transcript information unique to each cell to characterize cellular identity and function and to understand and identify the most critical cellular biological processes in complex tissues. It can also be used to further analyze the key factors necessary for the development of reproductive organs, stem-shoot meristematic tissues, and other organs, and to provide a theoretical basis for the cultivation of superior varieties. For example, in 2019, Zhang et al. performed scRNA-seq of 85 *Arabidopsis* root tips (0.5 cm-long apical regions) using *Arabidopsis* root tip tissues and classified the 7695 root tip cells obtained into 24 cell clusters. Rare cell types were identified through cellular annotation, revealing the heterogeneity of *Arabidopsis* root tip cells, and many cell type marker genes were found, which enabled construction of a high-precision developmental trajectory map of *Arabidopsis* root cells, realizing the precise localization of cell division and differentiation of the root meristem at the single-cell level [77].

### 5.2. Constructing Cell Dynamic Developmental Trajectories

In many biological processes, the organism responds to a variety of environmental stimuli with cells transforming in different states. Remodeling the process of cellular change over time is performed by constructing the trajectories of change between plant cells, digging deeper into the alteration of their cell types as the cell state changes. This can further resolve cell differentiation pathways, understand the dynamic developmental processes of plant cells, and reveal dynamic molecular features of the developmental landscape. It can reveal new dimensions for resolving regulatory factors necessary for organ development, resolving cellular heterogeneity, and spatial location molecular interactions. Mapping spatial transcriptional expression profiles of individual cell layers across species and organs can reveal spatial conservatism and differentiation in biogenetic evolutionary processes. Evolutionary origins can be explored based on convergent evolutionary directions of organ tissues, cellular evolutionary trajectories, and spatial gene expression differences in plants of homologous or dissimilar origins. For example, scRNA-seq of protoplasts isolated from the cotyledons of 5-day-old *Arabidopsis* seedlings yielded 12,844 cells clustered in 11 cell clusters, and the analysis of mimetic developmental trajectories of marker gene expression using Monocle2 constructed different developmental processes of stomatal spectrum cells, revealing the potential interactions among them. This study provides a new research idea for exploring the chronotropic trajectories and regulatory mechanisms of cell development in aboveground plant tissues [22].

### 5.3. Studying the Regulatory Networks of Different Transcription Factors

Coordinated plant growth and development and signaling in response to external stimuli require efficient signal exchange between tissues as well as cells. Different signal transducers are regulated by transcription factors in response to different changes. High-resolution analysis of photosynthesis, respiration, transpiration, distal transport, healing regeneration, rhizobial infection, maturation, and accumulation in various research models has involved the regulation of cell types and spatial molecular features and mining of new molecular markers and transcriptional regulators. For the study of the regulatory mechanism of plant development, analyzing the regulatory network of transcription factors starting from different cell types helps to deeply understand the biological function of cell development. During coordinated plant growth and development, transcription factors play a central role in cell fate decisions. In 2019, Denyer et al. performed single-cell sequencing of *Arabidopsis* root tip protoplasts, obtaining transcriptome data from a total of 4727 single cells, which were analyzed by mimetic timing to clarify the core genes associated with developmental stages and their inter-regulatory roles. These core transcription factors play a wide range of root growth and differentiation. The study constructed a gene regulatory network and also revealed the existence of a complex regulatory network between early differentiation initiation and terminal differentiation processes [18]. SnRNA-seq *Mesembryanthemum crystallinum*, collected at dawn and dusk for the transition between C3 and sedum acid metabolism (CAM) and data analysis revealed substantial transcriptional changes in chloroplasts at the onset of CAM induction, and cell trajectory extrapolation analyses reconstructed a 24 h CAM and C3 cycle with different expression patterns of key biological clock genes in CAM and C3 cell trajectories, pointing to a link between circadian regulation and CAM induction [78]. Analysis of anthropomorphic trajectories of *Fragaria vesca* leaves infected with *Botrytis cinerea* revealed signals for the transition of epidermal and sclerotial cells of strawberry leaves infected with gray mold from normal functioning to a defense response, with disease resistance-related genes being expressed in different patterns in different cell types, with disease resistance-related genes and genes encoding transcription factors are highly expressed in sing-cell types and interact to trigger systemic immunity of plants against *B. cinerea* [79].

### 5.4. Exploring the Response to Stress by Different Cell Types

scRNA-seq can be used to uncover the characteristics of cell types and spatial molecules involved in the regulation of various stress response processes and reveal the response mechanisms of cell types and spatial molecules to stress. It can be used to explore the mechanism of stress resistance based on the differences in gene expression in different tissue regions and cell types in response to biotic and abiotic stresses. Abiotic stress is an important environmental factor affecting plant growth and development, and the identification of cell types and changes in the composition of cell populations under abiotic stress can be determined through sequencing of single cells to understand the mechanisms of plant cells and developmental biology at the single-cell level. For example, in 2020, Wang et al. performed scRNA-seq of wild-type rice and rice seedlings under high salt stress, yielding a total of 4580 cells, with five cell types identified in the wild-type versus the control group. Further analysis of the differential genes between the different samples revealed that abiotic stress-induced changes in the transcriptome varied according to cell type. In addition, abiotic stress treatment also altered the composition of cell populations and slowed down the differentiation of chloroplasts. Analysis of rice cells and their developmental responses to abiotic stresses using single-cell transcriptomes can provide a better understanding of the changes in the cell types in the aboveground portion of rice seedlings grown in various environments, providing support for favorable resources for the discovery of the biology of the pusher plant [80]. In 2020, Wendrich et al. studied low phosphorus-induced root epidermal cell proliferation in *Arabidopsis* and found that the target genes of the *TMO5/LHW* complex were mainly enriched in root hair cells, suggesting that *TMO5/LHW* plays a role in the development of root hairs. The *tmo5* mutant exhibited normal root hair density, whereas overexpression of *TMO5* and *LHW* resulted in a significant increase in root hair density. Low phosphorus significantly increased *Arabidopsis* root hair density, whereas the *tmo5/tmo5-like1* double mutant showed no change in root hair density under low phosphorus conditions, suggesting that the increase in root hair density under low phosphorus was dependent on *TMO5* function [81]. ScRNA-seq of heat stress-treated cabbage leaves and stems identified 19 cellular subpopulations and seven major cell types, also revealing that heat stress affects not only the cell type-specific manner of gene expression but also the predominance of subgenomes [82].

## 6. Challenges of ScRNA-seq and Spatial Transcriptome Sequencing Technologies in Plants

### 6.1. Challenges in Sequencing Plant ScRNA-seq

The application of scRNA-seq in plant research faces several challenges [83]. First, plants contain cell walls, leading to technical difficulties in performing protoplast extraction and isolation in leaves or other organs of many plants, which may artificially cause changes in the expression of hundreds of genes and affect the ensuing transcriptome analysis [51]. Also, the cell walls of plants contain complex polysaccharides that must be permeabilized or removed before single-cell analysis, which is a key challenge that hinders the potential application of single-cell technology in plant species [84]. The isolation of protoplasts can vary in different plants or different tissues of the same plant, limiting the prevalent use of enzymatic methods [85]. Secondly, annotation of cell types is equally challenging, with non-conservation of the function of homologous genes between species significantly affecting the accuracy of cellular annotation, with extensive variation in marker genes between species as well as between different tissues of the same species or within the same tissue [86]. In addition, many cell types are resistant to protoplasts, which may produce biases in cell type proportions [82]. Thirdly, tissue dissociation leads to loss of cellular spatial information, which is not only important for the analysis of cell–cell and cell–environment interactions and for assigning specific functions to cells in the correct physical location, but it is also critical for cell type identification, especially for cell type-specific genes without known markers. Due to these limitations of scRNA-seq in plant research, the development of techniques for analyzing gene expression with transcript localization information is highly desired.

### 6.2. Challenges for the Plant Spatial Transcriptome

Plant cells have two major differences compared to animal cells, cell walls and large vesicles, which are also important reasons that prevent applying spatial transcriptomes in plants. Plant cells use the cell wall as a support, as the rigidity is more than enough but not too flexible. Plant cells are neither extremely thick nor thin when sectioning and are easy to break or detach from the embedding agent in frozen sections. The large vesicles of plant cells increase the cell volume but also increase the water content of plant cells, easily forming sharp ice crystals in frozen-embedded cells, thus destroying the integrity of the section and resulting in poor sectioning. The mRNA content per unit area also decreased, resulting in low transcript levels in the sections [87]. Most spatial transcriptome studies are based on young tissues characterized by thin cell walls that are easy to freeze and embed, whereas tissues with a high degree of lignification may be damaged during processing, which may compromise the integrity of reverse transcription products and affect subsequent analyses. In addition, the difficulty of RNA permeabilization of plant tissues is increased due to the varying degrees of structural lignification and low levels of RNA content, and young tissues are required to obtain sufficient RNA, resulting in the study of the spatial transcriptome in woody plants being a difficult task. Therefore, the establishment of pan-tissue single-cell atlases that cover different tissues, developmental stages, and environmental conditions of woody plants will significantly enhance the understanding of cell type diversity and provide new perspectives for studying the evolution and origin of specific traits in woody plants [86].

## 7. Expectations

Single-cell transcriptome analysis can classify cell types throughout plant tissues, while spatial transcriptome analysis can obtain spatial information about the entire tissue. Combining scRNA-seq and spatial transcriptome techniques can cover a wider range of cell types and also provide new insights into the logic of regulation and spatial organization between cells [88]. Performing comparative transcriptome analyses provides valuable insight into evolutionary and developmental relationships between cell or tissue types of different species. The development of single-cell transcriptomics techniques has opened up new possibilities for studying cell type inheritance and inferring cell type-specific evolution. However, one of the drawbacks of single-cell transcriptomics is that it destroys information about the spatial location of tissues. The advent of spatial transcriptomic technologies has addressed this limitation by enabling the precise localization of various cell types within plant tissues and facilitating the mapping of gene expression in different tissue regions. Integrating scRNA-seq/snRNA-seq with spatial transcriptomics holds great promise as a method to elucidate spatial expression patterns within plant tissues. This technology will facilitate clear visualization of detailed transcriptomic gene expression analysis in tissues, leading to an understanding of the spatial resolution and location of cell types and clarifying the role of intercellular interactions and cellular co-localization.

The most significant difference between single-cell transcriptomics and spatial transcriptomics is that the former has high resolution but loses spatial information, whereas the latter retains spatial information but is unable to achieve precision. Most scRNA-seq studies have been conducted in model plants and other plant species remain largely unexplored, while spatial transcriptomes have been less studied in plants. Looking forward, comparative studies of single-cell transcriptomics and spatial transcriptomics data across plant species could provide new insights for the discovery of additional cell types [88] and cell type specificity of complex traits [89]. In the future, more effort in single-cell transcriptomic technologies is needed to increase the applicability of protoplast release methods, and more effort in spatial transcriptomic technologies is needed to achieve high resolution. More importantly, the combined application of single-cell transcriptomics and spatial transcriptomics needs to be explored to create ample possibilities for research.

However, single-cell and spatial sequencing still faces many challenges. Currently, most single-cell and spatial transcriptome research is focused on model plants, and many challenges remain in non-model plants. Cell type identification is performed using marker genes from model plants, but in many species, marker genes from model plants are not sufficient to accurately identify cell types in non-model species. In order to locate marker genes, cross-species homologous gene searches can be performed, but it was found during homologous gene searches that homologous gene conversion is not possible in some species, which makes the identification of cell types in non-model plants more difficult, and for this reason, more and more marker gene identification methods have been developed. In previous studies, the localization of marker genes in identified cell types was mostly verified by in situ hybridization. With the increase in the number of species studied, when the cell type cannot be determined and cell type determination can be performed by in situ hybridization, gene localization is performed first, and the cell type is identified according to its function to determine the new marker genes in a specific cell type. With the development of technology, single-cell and spatial sequencing technologies are also gradually being applied to various tissues of non-model plants, and new marker genes are being discovered. More and more technologies are being researched and developed, and a number of difficulties in single-cell and spatial sequencing are being solved. The discovery of more new marker genes and their roles at the single-cell level, as well as the effects of external factors on plant growth and development, will contribute to the exploration of more plant candidate genes and their utilization to improve crop traits and the development of more synthetic genome breeding, which will provide new tools for crop development.

## Figures and Tables

**Table 1:** Partial research progress of plant scRNA-seq/snRNA-seq and spatial transcriptome sequencing in plants.

Study Species	Research Site	Research Method	Research Direction	References
*Arabidopsis thaliana*	root	ScRNA-seq	developmental differentiation locus	[17,18,19]
	root		phosphate stress	[20]
	stem		cell types and developmental trajectories	[21]
	leaf		stomatal/vein development	[22,23,24,25]
	seed		endosperm transcription map	[26]
*Oryza sativa*	root		gene regulatory network/developmental trajectory	[27,28]
	inflorescence		differentiation trajectories and regulatory networks	[29]
	seedling		abiotic stress response mechanism	[30]
*Brassica rapa* var. *glabra*	leaf		cell differentiation locus	[31]
*Zea mays*	Stem (SAM)		development of stem cells	[32]
	leaf		developmental regulatory network	[33]
	anther		meiosis	[34]
	ear		gene regulatory network	[35]
*Phyllostachys edulis*	basal root		developmental trajectory	[36]
*Lotus corniculatus*	root		cell type and function	[37]
*Populus*	stem		developmental trajectories and regulatory networks	[38,39]
*Arachis hypogaea*	leaf		developmental trajectories and regulatory networks	[40]
*Manihot esculenta*	leaf		developmental trajectories and photosynthesis	[41]
*Camellia sinensis*	leaf		gene expression network	[42]
*Hevea brasiliensis*	leaf		cell-specific gene expression patterns	[43]
*Taxus wallichiana* var. *chinensis*	leaf		gene regulatory network	[44]
*Fragaria* × *ananassa*	leaf		aingle-cell mapping after infection	[45]
*Nicotiana tabacum*	corolla		single-cell metabolic heterogeneity	[46]
*Bombax ceiba*	capsule		cell developmental trajectories and regulatory networks	[47]
*Camellia oleifera*	ovule		gene regulatory network	[48]
*Gossypium*	ovule		gene regulatory network	[49,50]
*Solanum lycopersicum*	stem	SnRNA-seq	spatial and temporal development trajectory	[51]
*Arabidopsis thaliana*	seed		construction of cell map	[52]
*Mesembryanthemum crystallinum*	leaf		gene expression pattern	[53]
*Arabidopsis thaliana*	leaf	Spatial transcriptome sequencing	spatial expression patterns of genes	[54]
	inflorescence		spatial development characteristics	[55,56]
*Populus tremula*	leafbud		spatial development characteristics	[55,56]
*Picea abies*	female cone		spatial development characteristics	[55,56,57]
*Solanum lycopersicum*	fruit		spatial development trajectory	[58]
	callus		gene regulatory network	[59]
*Brassica rapa* var. *glabra*	leaf		gene regulatory network/heat stress	[60]/[61]
*Portulaca oleracea*	leaf		spatial gene expression patterns	[62]
*Phalaenopsis aphrodite*	flower		spatial and temporal trajectory of development	[63]
*Populus*	stem		gene regulatory network	[64]
*Arabidopsis thaliana*	leaf	ScRNA-seq + single-cell transcriptome	spatial development trajectory	[65]
*Populus*	stem		gene regulatory network	[66]

**Table 2:** Key genes of various cell types in different tissue classes.

Organization Type	Cell Type	Key Gene	References
root	root cap	*SMB*, *BRN1*, *PH02Gene15627*, *Lj1gvBRI21766*	[19,36,37]
	root hair	*PRPL1*, *EXPA7*, *Lj1gvBRI21766*	[28,37]
	endodermal cells with casparian strip	*AT3G22120*	[18]
	quiescent center	*TEL1*	[18,19]
	meristem	*WOX5*, *PIN1*, *RGF3*, *PLT1*, *IAA33*, *ARF5/10*, *YUC3/8/9*, *Os03g0155500*, *OsGATA6*, *OsERF108*, *OsBUL1*	[17,28]
	meristematic vasculature	*PIP2-8*	[18]
	xylem	*VND7*, *Lj1gvBRI17778*, *Lj1gvBRI11791*	[19,37]
	phloem	*PME32*, *APL*	[18]
	xylem pole pericycle	*XPP*	[19]
	procambium	*SHY2*	[19]
	endodermis	*MYB36*	[19]
	cortex	*CORTEX4*, *AT1G62500*, *Lj1gvBRI10561*	[18,19,37]
	epidermal cell	*PH02Gene14566*, *Lj1gvBRI08110*	[36,37]
	trichoblast	*COBL9*, *MES15*, *Os10g0578200*	[18,19,28]
	atrichoblast	*GL2*, *MLP34*, *Os01g0248900*	[18,19,28]
	pericycle cell	*ATL75*	[18]
apical meristem of the stem	epidermal cell	*SlML1*, *SlPDF2*, *SlPDF1*, *SlFDH*, *SlFLP1*, *SlPEL3*, *ATML1*, *PDF1*, *FDH*	[21,51]
	mesophyll cell	*CRR23*, *RBCS2B*, *PNSL1*	[21]
	shoot meristematic cell	*STM*, *KNAT1*, *KNAT2*, *KNAT6*	[21]
	SAM cells	*SlSTM*, *SlBP*	[51]
	proliferating cell	*HIS4*, *CDKB2;1*, *CYCA1;1*	[21]
	vasculature cell	*SIDOF2.1*	[51]
	xylem	*SlPXY*, *SlPHB*, *SlCNA*, *SlLHW*, *SlMP*, *SlREV*, *PXY*	[21,51]
	phloem	*SlPP2-A1*, *SlPP2-B12*, *SMXL5*	[21,51]
	guard cell	*FMA*, *EPF1*, *POLAR*, *BASL*, *TMM*	[21]
	companion cell	*PP2-A1*, SUC2	[21]
	trichome cell	*SlANL2*, *SlMX1*	[51]
	leaf primordium adaxial and abaxial cell	*SlLHCA2*, *SlCAB1*	[51]
stem	epidermal cell	*GL2*, *KCS10*	[66]
	vessel element	*PagSCP*, *PagASP1*, *PagAEP3*, *PagXCP1*, *PagXCP2*, *PagMC9*, *PagEXP6*, *PagLAC12*	[38]
	fiber cell	*PagCAld5H2*, *PagTUB8*, *PagLAC12*	[38]
	xylem parenchyma cell	*SND1*, *NST1*	[39]
	phloem cell	*APL*, *PP2-A1*, *PP2-A4*, *PP2-A10*, *SEOR1*, *CLE41*	[39,66]
	phloem parenchyma cell	SUC2, *SEOR1*, *UMAMIT9/12/20/21/22*	[39]
	xylem mother cell	*PtrHB4*, *PtrHB7*	[39]
	phloem mother cell	*OPS*, *SMXL5*	[39]
	cortex/endo dermis initial cell	*SCR*, *SCL23*, *CYCD6;1*	[39]
	cortex and pith	*CCL*, *PSBX*	[66]
	endodermal cell	*NPF3.1*	[39]
	cambium cell	*WOX4*, *PXY*	[66]
	xylem precursor	*ACL5*, *LOG3*, *MP*, *PHB*	[66]
	xylem fiber	*SND1*, *MYB103*	[66]
	phloem precursor	*ANT*, *BAM3*	[66]
	phloem parenchyma	*UMAMIT12*, *UMAMIT19*, *UMAMIT22*	[66]
	vessel cell	*MAN6*, *VND1*, *XCP1*, *XCP2*	[66]
	sieve cell	*Cals7*, *APL*, *SEOR1*	[66]
leaf	pavement cell	*RBCS*, *IQD5*, *RBCS1A*	[22,44]
	guard mother cell	*BASL*, *EPF1*, *SCRM/2*, *FAMA*, *HIC*	[22]
	guard cell	*FAMA*, *HIC*, *RBCS*, *EPF1*, *MPK12*, *TMM*, *SPCH*, *SCAP1*, *FLP/MYB124*, *KCS1*, *KCS20*, *MYB60*, *MAPK9*	[22,23,25,41,44]
	stomatal complex cell	*FLP*, *GL2*	[44]
	meristemoid mother cell	*SPCH*, *POLAR*, *EPF2*, *TMM*, *MUTE*, *HDG2*	[22]
	meristemoid	*SDD1*, *BASL*, *MUTE*, *EPF1*	[22]
	phloem	*SMXL5*, *SWEET11*, *PP2LA9*, *APL*	[25,42,44]
	phloem parenchyma cell	*APL*, SEOB, *SWEET11*, *SWEET12*, *IRX7*, *CDF4*, *SULTR2;1*, *BZIP9*, *DOF5.6*	[23]
	companion cell	SUC2, *FTIP1*, *HIPP36*, *AHP1*, *TET6*, *PHL12*, NAKR2	[23,41]
	bundle sheath	*SCL23*, *SULTR2.2*	[23]
	epidermis cell	*ATML1*, *CUT1*, *KCS1*, *FDH*, *KCS20*, *PAL*, *4CL3*, *CCR1*, *CAD1*, *CAD9*, *pod*, *GLIP*, *CER1*, *PDF1*, *ABCG11*, *SVB2*, *CER3*	[23,41,42,43,44]
	adaxial epidermis	*CHS*	[41]
	vascular cell	*LAC17*, *LAC6*, *LAC14*, *COMT*, *ACL5*, *SUC2*, *PXY*	[41,43,44]
	xylem	*TMO5*, *PXY*, *VND7*, *ACL5*, *PIN6*, *Nek2*, *KIN14N*	[25,41,42]
	xylem parenchyma cell	*ACL5*, *GLR3.6*, *AGP31*, *CEL1*, *CESA8*, *COBL4*, *SDH*	[23,24,41]
	hydathode	*PUP1*, *EP3*, *NHL1*	[23]
	mesophyll cell	*LHCB2.1*, *RBCS1B*, *SWEET11*, *LHCB4.1*, *LHCB5*, *RBCS*, *CAB7*, *LHCB4.2*, *LHCB6*, *CAB3*	[23,41,43,44]
	spongy mesophyll cell	*YAB1*, *F3H-3*, *FLS*, *ANS*, *ANR*, *PDF1*	[41,42]
	palisade mesophyll cell	*RBR*, *FDH3*, *TGLIP*, *IRS*, *RD22*, *DCR*	[41,42]
	sieve tube element	*DOF2.4*	[23]
	xylem parenchyma cells with features relating to xylem differentiation	*REV*, *ATHB-15*, *HB-8*, *REV/IFL1*, *PHB*, *CNA/AtHB15*	[23,24]
	procambium cells with features relating to phloem differentiation	*BAM3*, *CLE45*, *CVP2*, *CVL1*, *APL*, *CLE41*, *DOF5.1*, *LBD3*	[23,24]
	procambium	*TMO5/BHLH32*	[25]
	sieve elements	SEOB	[41]
	cambium cell	*WOX4*, *TDR*	[41]
flower	floret	*Trps8*, *AP2/ERF*, *OsMADS1*, *DL*, *YABBY*	[29,34]
	spikelet meristems	*BD1*	[35]
	adaxial meristem periphery domain	*BA1*, *ZmPAO1*, *ZmCUC3-LIKE*	[35]
	lateral organ epidermis domain	*LTP1*	[35]
	adaxial domain	*DRL2/ZmYAB7*	[35]
	abaxial domain	*ZmARF3/4-LIKE1*	[35]
	meristem base	*RAMOSA*	[35]
	meristem epidermis cell	*ZmHDZIV8*	[35]
	xylem cell	*ZmMYB46*, *ZmXCP2*, *ZmTMO5-LIKE3*, *ZmTMAAT*, *ZmWAT1*	[35]
	phloem cell	*ALTERED PHLOEM DEVELOPMENT*, *ZmZNF30*	[35]
	sieve element	*PEAR1/PEAR2*	[35]
	companion cell	*PHLOEM PROTEIN 2-LIKE A1*	[35]
	bundle sheath	*ZmSHR1*	[35]
	cortex	*ZmNPF6.4-LIKE2*	[35]
	pith cell	*ZmNAC122*	[35]
	epidermal cell	*MALE FLOWER SPE CIFIC 18*, *ZmPLTP3*, *ECERIFERUM4*	[35,46]
	dividing cell	*ZmCYCB2-4*, *ZmHIS2A*	[35]
	spikelet	*OsMADS1*	[29]
	flower cell	*DWT1*, *OsMADS6*, *OsMADS8*	[29]
	lemma	*OsMADS1*	[29]
fruit/seed	chalazal cyst	*AT2G44240*, *AT4G13380*	[26]
	chalazal nodule	*AT5G10440*, *AT1G44090*	[26]
	initiated fiber cell cluster	*HD1*, *BZR1*, *MYB2*, *TRY*, *HOX1*	[47]
	epidermal cell	*HIC*, *CoAKR*, *CoCYP81E8*, *CoUGT73C5*, *CoUGT91A1*	[47,48]
	synergid cell	*CoECA1*	[48]
	procambium cell	*CoDOF4.6*	[48]
	fibre cell	*MYB25-like*, *MYB25*, *MML9*, *HOX3*, *XTH*	[49,50]
	ovule	*ToFZY2*, *ToFZY3*, *Solyc03g112460*, *PIN5*, *ARF7*, *ARF9*, *IAA14*, *IAA35*	[58]
	ovary wall	*ToFZY1*, *ToFZY5*	[58]
	ovary wall/pericarp	*ToFZY5*, *PIN6*, *PIN7*, *PIN1*, *LAX3*, *ARF9*, *IAA17*	[58]
	pericarp	*ToFZY1*	[58]

## Data Availability

Not applicable.
